# Mid-level perceptual features, and not ambiguity, accelerate access to awareness

**DOI:** 10.1093/nc/niag006

**Published:** 2026-02-20

**Authors:** Nadav Amir, Uri Maoz, Liad Mudrik

**Affiliations:** School of Psychological Sciences, Tel Aviv University, Faculty of Social Sciences, Chaim Levanon Street 30, Ramat Aviv, Tel Aviv 69978, Israel; Princeton Neuroscience Institute, Princeton University, 40 Woodlands Way, Princeton, NJ 08540, United States; The Fields Institute for Research in The Mathematical Sciences, 222 College Street, Toronto, Ontario, M5T 3J1, Canada; Schmid College of Science and Technology, Chapman University, 1 University Drive, Orange, CA 92866, United States; Institute for Interdisciplinary Brain and Behavioral Sciences, Chapman University, 14725 Alton Parkway, Suite 200, Irvine, CA 92618, United States; Crean College of Health and Behavioral Sciences, Chapman University, One University DriveOrange, CA 92866, United States; Anderson School of Management, University of California Los Angeles, 110 Westwood Plaza, Los Angeles, CA 90095, United States; Biology and Bioengineering, California Institute of Technology, 1200 E. California Blvd., Pasadena, CA 91125, United States; School of Psychological Sciences, Tel Aviv University, Faculty of Social Sciences, Chaim Levanon Street 30, Ramat Aviv, Tel Aviv 69978, Israel; Sagol School of Neuroscience, Tel Aviv University, P.O. Box 39040, Ramat Aviv, Tel Aviv 69978, Israel; Canadian Institute for Advanced Research (CIFAR), Brain, Mind, and Consciousness Program, 661 University Ave., Suite 505, Toronto, ON M5G 1M1, Canada

**Keywords:** continuous flash suppression, observability, symmetry, ambiguity

## Abstract

Current theoretical accounts of perception and high-level cognition suggest that awareness plays an active role in disambiguating incoming sensory information. However, the relationship between ambiguity resolution and conscious access remains unclear, partially due to a lack of quantifiable measures of ambiguity. Here, we describe a novel paradigm designed for testing whether more ambiguous stimuli would enjoy preferential access to awareness, as indexed by the time it takes them to break interocular suppression in the breaking continuous flash suppression paradigm. In a series of three experiments, we found that stimuli’s mid-level perceptual features (most likely, visual symmetry levels), rather than their ambiguity, facilitated access to awareness. We therefore propose that such features can drive preferential access to awareness and hypothesize that the potential effect of symmetry might be driven by information redundancy due to the invariance of symmetric patterns under geometric transformation.

## Introduction

Ambiguity is ubiquitous. As you walk down a busy street, you are bombarded by a constant barrage of sensory signals that are ambiguous at face value, but are nevertheless quickly interpreted by your brain and emerge into your awareness as meaningful objects: people, cars, trees, etc. Ancient Indian philosophers invoked the image of a coiled rope, which in poor lighting may appear to be a snake, as a metaphor for the ambiguous and illusionary nature of our perception (Rao 2011). Similarly, modern Bayesian accounts of perception suggest that the content of our awareness reflects the outcome of a subliminal inference—an ambiguity resolution process that determines the most probable hidden causes underlying the raw stream of information impinging upon our sensory systems (Kersten et al. 2004). This notion harks back at least to Helmholtz’s view of perception as unconscious inference (Von Helmholtz 1910). Under this view, possible interpretations of incoming ambiguous signals are subliminally evaluated, and the most likely one is granted access to our awareness. While the notion of perception as unconscious inference has gained influence within consciousness research (Hohwy et al. 2008, Hohwy and Seth 2020, Panichello et al. 2013, see also Dehaene 2011), few empirical studies have focused on quantifiably measuring the effect of semantic ambiguity on conscious access.

Previous studies have used the continuous flash suppression (CFS) paradigm (Tsuchiya and Koch 2005), to test whether different stimuli features modulate their access into awareness (for reviews, see Gayet et al. 2014, Pournaghdali and Schwartz 2020, Stein 2019). In the CFS paradigm, multiple visual patterns (called Mondrians) are flashed successively at a rate of ~10 Hz to one eye only. This stimulation can reliably suppress the conscious awareness of the image presented to the other eye for an extended period, lasting between 2 and 10 seconds on average. Studies using CFS often employ a technique referred to as breaking-CFS (b-CFS), where shorter emergence times are thought to represent prioritized access to consciousness (Stein et al. 2011, Stein 2019).

Thus far, b-CFS has only been used indirectly to study the potential effect of ambiguity on access to consciousness. In particular, subjective surprise levels, which have been shown to affect b-CFS emergence times, may be interpreted as related to the ambiguity of the stimulus and the ease by which it can be processed. For example, one study reported that stimuli that are more familiar, such as upright versus inverted faces or familiar versus unfamiliar words, emerge faster into awareness (Jiang et al. 2007). In another study, statistically predictable stimuli have been shown to emerge into awareness faster than non-predictable ones (Pinto et al. 2015), and a similar result was obtained for stimuli that were semantically primed (Lupyan and Ward 2013). Taken together, these studies suggest that the degree of familiarity, or the predictability of a stimulus, might affect its ability to break suppression and emerge into awareness.

Such an interpretation is consistent with Bayesian (Yuille and Kersten 2006) and predictive processing (Hohwy et al. 2008) accounts of perceptual awareness, suggesting that stimuli that conform with prior expectations, whether formed by long-term familiarity or proximate priming, may be more readily processed and perceived (de Lange et al. 2018). Importantly, predictability is often inversely related to ambiguity, since a predictable stimulus is one that can typically be inferred from its context with high confidence. For example, our familiar daily experience allows us to unambiguously identify the bright yellow disk rising from the east each morning as the sun. In contrast, the precise shape of a cloud is often unpredictable and interpretable as various familiar objects, such as an animal or a face, resulting in a highly ambiguous percept. Could it therefore be that predictable and familiar stimuli enjoy preferential access to awareness because they are perceived as less ambiguous? Conversely, could ambiguous stimuli enjoy preferential access to awareness since they can be more easily interpreted as familiar and predictable objects?

Our focus in this study is on epistemic ambiguity, namely uncertainty regarding the semantic interpretation of the stimulus that stems from incomplete sensory information. Epistemic ambiguity can be contrasted with perceptual ambiguity (Wang et al. 2013, Rodríguez-Martínez et al. 2018) that is exhibited by stimuli that are fully specified but support multiple perceptual interpretations, such as the Necker cube (Kornmeier and Bach 2005a) and other bistable images (Schwartz et al. 2012). Unlike perceptual ambiguity, resolving epistemic ambiguity requires inferring or completing additional information that is *not contained in the stimuli*. In many cases, disambiguation is only enabled *via* another stimulus, which provides a constraining context. Though this epistemic ambiguity has not yet been studied with respect to emergence into awareness, previous studies testing whether resolution of such ambiguity requires consciousness yielded conflicting results. One study suggested that such inference can occur unconsciously (Chang et al. 2016), yet more recent work supports the notion that it requires awareness (Meijs et al. 2018, Alilović et al. 2021, Tal et al. 2024), at least when non-trivial semantic integration is involved (Biderman et al. 2020).

Here, we aimed to directly test whether the epistemic ambiguity of a stimulus affects the time it takes it to emerge into awareness, using ambiguous images of occluded dice faces. This test cannot speak to the controversy around unconscious resolution of epistemic ambiguity (since b-CFS might be driven by later processes; Stein et al. 2011). However, it does allow one to determine if such ambiguity enjoys a preferential status in emerging to awareness; if so, this could support claims that consciousness is needed to resolve epistemic ambiguity, when one possible interpretation out of many that are unconsciously evoked, is selected (Hohwy et al. 2008, Hohwy and Seth 2020, Panichello et al. 2013, see also Dehaene 2011).

**Figure 1:**
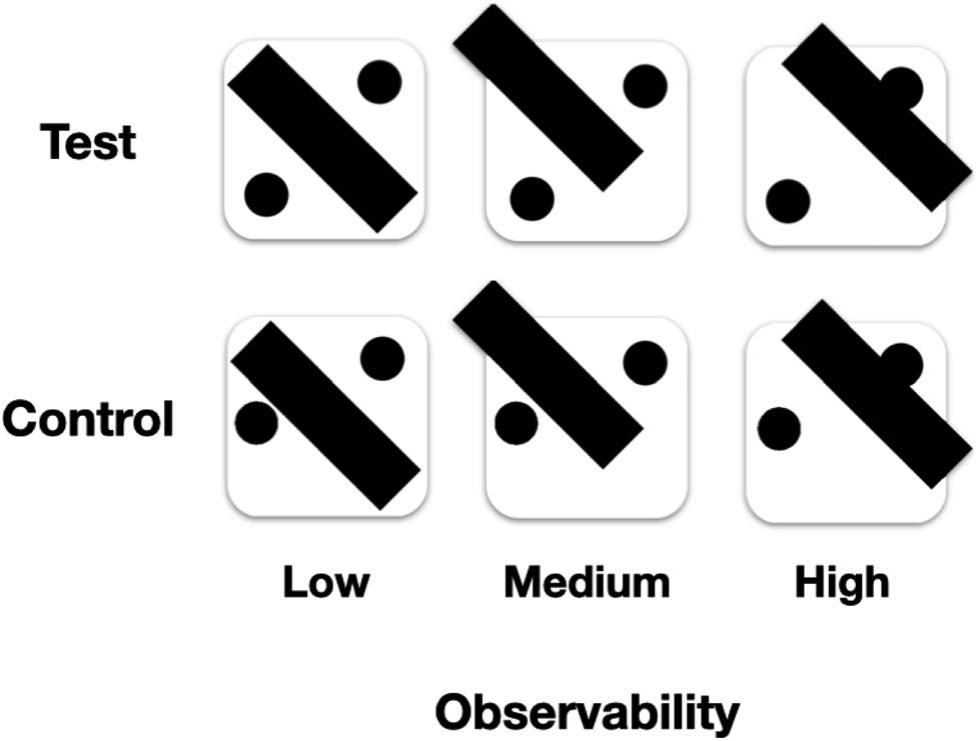
Experiment 1 stimuli: Test stimuli (top) were designed to visually resemble each other but have different observability levels by shifting the position of the occluding diagonal bar. The low observability stimulus could be interpreted as four possible dice configurations (2,3,4, or 5), the medium as two (2 and 3), and the high as a single one (2). Each control stimulus (bottom) was designed to visually resemble the corresponding test stimulus above it while lacking valid semantic interpretability as a standard dice configuration owing to the incongruent position of the dot on the left.

We generated a novel set of stimuli with three different epistemic ambiguity levels and measured how long it took each of them to break interocular suppression under CFS. To operationalize epistemic ambiguity, we drew on the control-theoretic concept of *observability*, defined as the amount of information that the observed outputs of a system provide about its potentially hidden internal states (Kalman 1963). The observability of a stimulus is thus inversely related to its ambiguity, since highly observable stimuli can be unambiguously associated to a single underlying cause, or hidden state. In the current setting, observations consisted of standard playing-dice faces, which were partially occluded such that the veridical number of the dots, or ‘pips,’ on the presented dice face could not be unequivocally determined, except for in the high observability conditions. In lower observability conditions, the visual stimuli could be interpreted in several ways, corresponding to dice configurations with different numbers of pips as illustrated in Fig. S1 in the supplementary. Hence, high observability corresponds to low epistemic ambiguity, or fewer configurations of pips that are logically consistent with the partially occluded dice image, and *vice versa* (Fig. 1, top).

To ensure that any differences in b-CFS latencies do not stem from differential processing of mid-level visual features of the stimuli, we also included control stimuli that were designed to be visually similar to the corresponding dice face images but lacked semantic interpretation in terms of possible dice latent states. In these stimuli, one of the pips was positioned at a non-congruent location with respect to standard playing dice (Fig. 1, bottom). Thus, though the mid-level features of the experimental and control stimuli were closely matched, the former were designed to evoke the interpretation of a standard dice face, with potentially occluded pips, while the latter were designed not to evoke such an interpretation. We accordingly predicted that these control stimuli would exhibit no correlation between “observability” (in quotes, since they have no meaningful latent state to infer) and b-CFS times, supporting the hypothesis that any main effect of observability in the test stimuli is driven by inference of the latent state of the dice rather than mid-level visual properties.

In a series of three pre-registered experiments, we tested whether observability levels affect b-CFS response times. Based on exploratory pilot data, we predicted that higher observability levels (or lower ambiguity) would inhibit access to awareness, as indexed by longer b-CFS latencies. The first experiment found evidence for such an effect in the test condition. However, a corresponding effect was also found for the control stimuli, despite the lack of meaningful observability level. To determine whether the effect was driven by mid-level visual properties of the stimuli, rather than observability, we conducted two additional experiments. These additional experiments revealed that mid-level properties of the stimuli (probably, their visual symmetry levels), rather than their observability, better explain the data: higher symmetry levels corresponded to shorter b-CFS response times, irrespective of observability. While our results did not support our original hypothesis, they instead highlight the role of mid-level perceptual factors, such as symmetry, in driving emergence to awareness, lending suggestive support to the well-established notion that visual symmetry facilitates conscious access (see further details in the Discussion section below).

## Experiment 1: observability and access to awareness

### Methods

#### Participants

Participants were 50 healthy volunteers (36 female), students in Tel-Aviv University aged 18–35 $\left(M=23.7, SD=3.4\right)$. Here and in the next two experiments, sample size was informed by simulations of a linear mixed effect model fitted to exploratory pilot data with 20 participants (14 female) aged 19–27 $\left(M=23.9, SD=2.6\right)$, indicating that a sample size of 50 would be sufficient to find a main effect of observability or an interaction effect of observability and condition, with a power level greater than 80% at a significance level of $\alpha =0.05$. All participants had normal or corrected-to-normal vision using contact lenses and reported no past neurological, attentional, or psychiatric disorders. All participants exhibited performance accuracy levels above the predefined exclusion threshold of 75%. Participants participated in the experiment for credit or payment; they signed a consent form and were clearly informed that they could withdraw from the experiment at any point, if they wished to do so. The experiment was approved by the Tel-Aviv University ethics committee and preregistered prior to data collection (https://osf.io/qp2j7/).

#### Stimuli and apparatus

Stimuli were presented on a CRT display (1 024 × 768, 100 Hz), through an adjustable mirror stereoscope attached to a chin rest and positioned in front of the display at a distance of 60 cm. MATLAB and Psychtoolbox 3 (Brainard 1997) were used to control stimuli presentation.

Stimuli consisted of black and white images of dice faces that were partially occluded behind a black diagonal bar that partially occluded some of the pips. Utilizing the familiar dot configurations of traditional six-faced playing dice, we designed three test stimuli, each exhibiting a different level of observability: low, medium, or high, corresponding to the amount of information they convey about the actual number of partially occluded dots on the presented face (Fig. 1, top). On high observability trials (right), the occluding bar was placed off-diagonally, making it possible to unambiguously identify the configuration as two pips, corresponding to full uncertainty resolution—from six possible configurations to a single one, translating into $\mathrm{lo}{\mathrm{g}}_2(6)\approx 2.6$ bits of information. On medium observability trials (middle), the configuration could be interpreted in two different ways: either as two or three pips, corresponding to an uncertainty reduction of $lo{g}_2\left(6/2\right)\approx 1.6$ bits. Finally, for the low observability stimulus (left), the occluding bar was positioned on the center diagonal such that the resulting image was consistent with four possible configurations, namely two, three, four, or five pips, evoking an uncertainty reduction of $lo{g}_2\left(6/4\right)\approx 0.6$ bits. Thus, higher observability corresponds to lower epistemic ambiguity and *vice versa*; more precisely, each level of observability corresponds to an increase of 1 bit of information, or ambiguity resolution, compared to the one below it.

#### Procedure

The experimental session consisted of 480 trials, composed of eight blocks of 60 trials each, with a short break in between. Before the main session, ocular dominance was determined using the Miles test (Miles 1930). Participants then received verbal and written instructions explaining the task, specifically framing the stimuli as dice that can have different numbers of pips. We explained that in some displays, the possible number of pips cannot be inferred unambiguously. Then, to make sure participants indeed understood the stimuli and interpreted them as intended, so to portray different levels of ambiguity, they performed a training block consisting of five trials. Then, they provided verbal confirmation that they had understood the task and were able to detect the dice stimuli. Before each session, participants were instructed to calibrate the display by adjusting the location of the frames on the screen until they achieved binocular fusion. Then, on half of the trials, the test (‘meaningful’) dice stimuli were presented (Fig. 1, top), with 80 trials for each of the three observability levels. On the other half of the trials, the control (‘meaningless’) stimuli were presented (Fig. 1, bottom), also with 80 trials for each one. Trial order was randomly intermixed between all six possible stimuli, including test and control. At the end of each session, participants were debriefed on the experiment. They were asked whether they used any heuristics that helped them detect the stimuli and whether they noticed anything unusual about them. None reported any change in their understanding of the stimuli.

**Figure 2:**
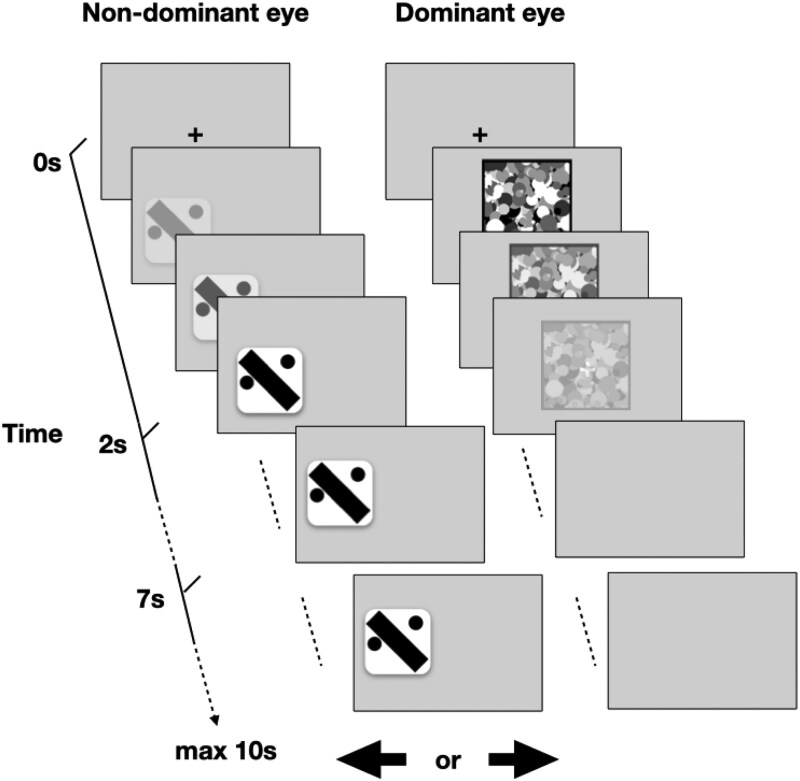
Experimental paradigm: Participant’s dominant eye was presented with a rapidly fluctuating pattern of grayscale circles of varying sizes (Mondrians), while the non-dominant eye was presented with a partially occluded dice stimulus. The contrast of the stimulus was gradually increased while that of the Mondrians decreased. The two visual streams were combined using stereoscopic glasses. Participants were instructed to press an arrow key indicating the side of the screen on which they saw the dice stimulus as soon it became visible. See methods for additional details.

Figure 2 describes the experimental procedure: Each trial started with a fixation cross presented to both eyes at the center of the screen. Participants indicated they were ready by pressing the spacebar, triggering the presentation of a single stimulus to the participant’s non-dominant eye either to the left or the right of the fixation position with equal probability. Flashing grayscale Mondrian patterns, consisting of 1000 circles with radii uniformly distributed between 3 and 30 pixels and luminance levels distributed uniformly over six equal intervals between black and white, were simultaneously presented to the participant’s dominant eye at a rate of 10 Hz. The dice stimuli were gradually faded in by linear contrast elevation from 0% to 100% over a period of 2 seconds, while the Mondrian patterns were simultaneously faded out by linear contrast reduction from 100% to 0% over a period of 7 seconds (so that the Mondrians continued to fade out for 5 seconds after the stimulus reached full contrast, becoming invisible 7 seconds after the beginning of the trial). Full details, including the code to run the experiment, stimuli, and collected data, are available online (https://osf.io/qp2j7/). Stimuli were shown for up to 3 additional seconds after the end of the 7-second fade-out period, hence when the dice stimulus was at 100% contrast and the Mondrians were at 0% contrast. Participants were asked to press the left or right arrow key as soon as they become aware of the stimuli at any time during the trial, to indicate whether they saw the stimulus appear on the left or right side of the fixation cross, respectively. Pressing one of the arrow keys terminated the trial.

#### Statistical analysis

In all three experiments, we fitted a linear mixed model (LMM) to analyze the effect of observability level on reaction time as well as the effect of the experimental condition (test or control) and the interaction between condition and observability. The dependent variable was log-transformed reaction time (in seconds). Fixed effects included trial condition (categorical: control versus test), observability (ordinal: low, medium, or high), and their interaction. To account for individual variability, random effects were specified for participants, including random intercepts and slopes for observability, trial condition and their interaction. The model was specified, using Wilkinson notation (Wilkinson and Rogers 1973), as follows:


$$ logRT\sim observability\ast condition+\left( observability\ast condition| subject\right), $$

and fitted using the fitlme function in MATLAB (version R2022a, The MathWorks, Inc., Natick, MA, USA). To evaluate the evidence for including specific fixed effects in the model, Bayesian Factors were approximated by comparing the Bayesian Information Criterion (BIC) of the full model to reduced models excluding each factor of interest. To detect differences between observability levels in each condition, reaction time data were also analyzed using a one-way ANOVA. Following significant results, the multcompare function in MATLAB was applied to the ANOVA-fitted object. Tukey’s Honestly Significant Difference (HSD) method was employed to test all pairwise differences across between observability levels, with p-values adjusted for multiple comparisons and significance set at $\alpha =0.05$. To adjust for multiple comparisons we used TreeBH, a procedure for controlling false discovery rate in hierarchically structured experimental designs (Bogomolov et al. 2021, Zeevi et al. 2025). The TreeBH adjustment is illustrated in Fig. S2 in the supplementary. All reported *P*-values below have been adjusted accordingly.

#### Results

**Figure 3:**
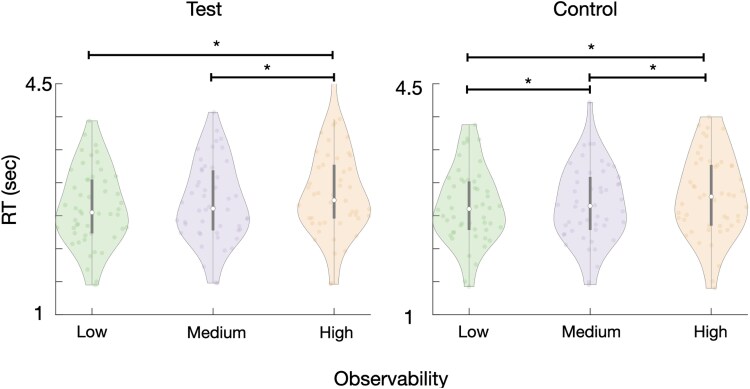
Experiment 1 results. Violin plots depicting reaction times (abscissa) as a function of observability level (ordinate) for test (left) and control (right) stimuli conditions. Each colored dot represents an individual participant, while the white dot along the axis of symmetry of the violin plot denotes the median across participants. The thicker vertical line around the median point represents the 25%–75% percentiles for each plot. Asterisks indicate significant differences between mean RTs for different observability levels at *P* < .05.

All participants performed at an accuracy level higher than the pre-registered inclusion threshold criterion of 75% ($M=97.5\%, SD=7.3\%)$. We fitted a linear mixed effects model, using log-scaled reaction times (RTs) as the response, with observability level (low, medium, or high; Fig. 1), trial condition (test or control) and their interaction as fixed effects, and participant as a random effect. The results are presented in Fig. 3.

The mixed effect model revealed a main effect of observability on RTs, such that longer RTs were associated with high observability trials versus medium and low ones, across both test and control conditions ($\beta =0.10, SE=0.01,t(23516)=9.45,P<.015$), corresponding to ~10.5% increase in RT per observability level. No main effect of trial condition was found ($\beta =-0.02, SE=0.01,t(23516)=1.28,P=.200$) and the interaction between observability and trial condition was also below significance ($\beta =-0.02, SE=0.01,t(23516)=1.65,P=.154$). A Bayes Factor analysis provided strong evidence against a main effect of condition or an interaction effect $\left( BF<0.001\right).$ Nevertheless, following the marginal significance, we ran *post hoc* comparisons using the Tukey HSD test, which showed that in the test condition, somewhat in line with our expectation, RTs were longer for the high observability stimuli $\left(2.83\pm 0.02\right)$compared with the medium $\left(2.69\pm 0.02\right)$ and the low $\left(2.63\pm 0.02\right)$ observability ones, which did not, however, significantly differ. However, contrary to our expectation, control condition trials also exhibited longer RTs for higher “observability” levels: with $\left(2.79\pm 0.02\right),\left(2.67\pm 0.02\right)$, $\left(2.64\pm 0.02\right)$ for the high, medium, and low “observability” trials, which also significantly differed from one another at the $\alpha =0.05$ threshold level.

#### Discussion

The effect of observability on RTs in the test condition was, by and large, consistent with our hypothesis that higher observability would be associated with delayed access to awareness. However, an interaction with trial condition was not found. In addition, post-hoc analyses showed that the effect was also found for the control stimuli.

These unexpected results might be explained in two different ways, both reflecting unintended confounds in the design: first, the similar behavior in the two conditions might stem from the mixed-trials within-participants design. Arguably, the intermittent exposure to the test and control stimuli could have primed participants into also interpreting the control stimuli as dice due to their visual resemblance to the corresponding test stimuli, especially under the non-ecological viewing conditions in CFS. If so, they might have processed them in the same way as the test stimuli, resulting in a similar effect of observability in both test and control trial conditions.

Alternatively, the results might be driven by lower-level visual properties of the stimuli, which were shared across both trial conditions, rather than by ambiguity processing. Specifically, the stimuli differed in their mid-level perceptual features. For example, the different positions of the occluding bar on the dice faces may have induced different levels of symmetry “gestalt” percepts, which have been previously reported to affect suppression times under CFS (Devyatko et al. 2019). In the low-observability condition, the positioning of the occluding bar along the center of the main diagonal of the dice face induced invariance under two symmetry axes: one for mirror reflection across the main diagonal and one for rotation in${180}^{{}^{\circ}}$. In the medium observability stimulus, however, the off-center position of the occluding bar along the main diagonal induced only a single symmetry, namely, mirror reflection along the main diagonal, while in the high observability stimulus, the off-diagonal position of the occluding bar did not induce any basic symmetry axes. Thus, it could be that this difference in symmetry evoked the observed results. Notably, the incongruent position of the pips in the control stimuli could have altered the perceived symmetry levels, introducing a potentially undesirable interaction between symmetry level and trial condition. However, the fact that the control condition showed similar results suggests that the perceived symmetry may be largely determined by the position of the bar, and not that of the pips.

To address both possible confounds, we ran an additional experiment with new control stimuli that (i) appeared only before the test stimuli using a block design and (ii) were modified to match the test ones in terms of symmetry level within each ambiguity level. We reasoned that if the effect is indeed driven by observability, it should be found only for the test stimuli, while if it is driven by symmetry, it should be observed in both conditions.

## Experiment 2: controlling for symmetry and priming effects

### Methods

#### Participants

Participants were 50 healthy volunteers (37 female), between the ages 18–35 $\left(23.6\pm 2.7\right)$. Performance accuracy for all participants was above the exclusion threshold of 75% ($M=97.4\%, SD=6.7\%)$. This experiment was also preregistered prior to data collection (https://osf.io/qp2j7/).

#### Stimuli and apparatus

**Figure 4:**
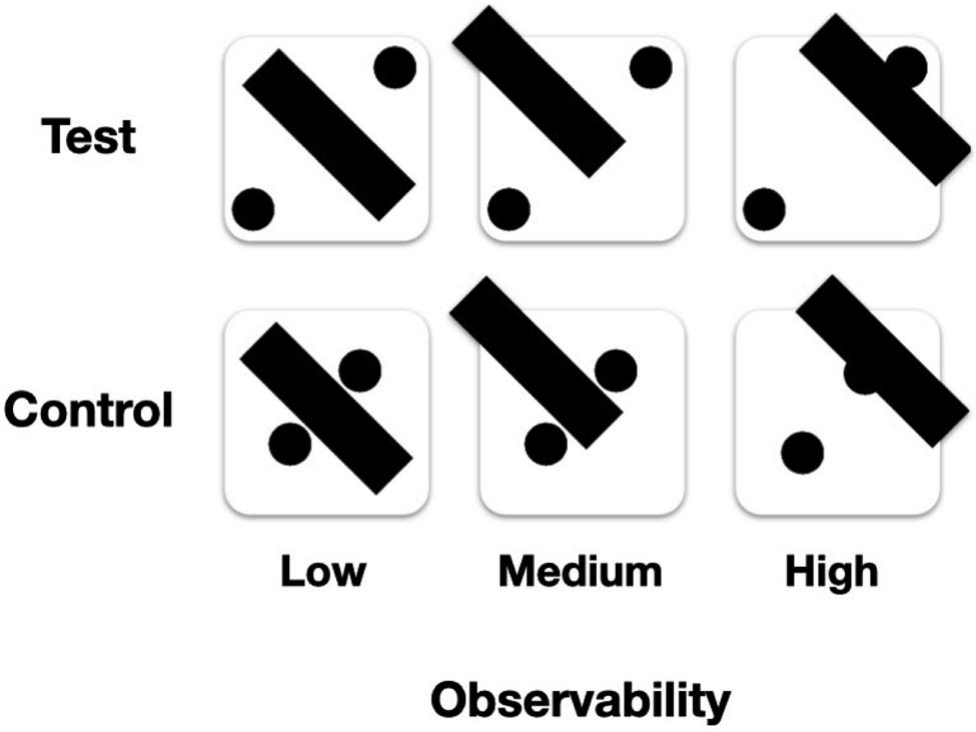
Experiment 2 stimuli: Test stimuli (top) were similar to those used in experiment 1, while control stimuli (bottom) were designed to resemble the corresponding test stimuli in terms of symmetry level, while lacking the same semantic interpretability.

The apparatus, the CFS stimulation, and the stimuli were similar to those used in Experiment 1. However, to maintain equal symmetry properties across conditions, the control stimuli in Experiment 2 were modified such that the two visible pips were placed on the same main diagonal as in the test stimuli, but in locations that are not characteristic of dice, near the center of the face, straddling the occluding bar from both sides (compare Fig. 4, bottom to Fig. 1, bottom). Furthermore, to mitigate possible confusion between the test and control conditions, the pips in test condition were shifted slightly toward the corners (compare Fig. 4, top to Fig. 1, top).

#### Procedure

The experimental procedure was similar to Experiment 1, with one important difference: to control for a possible priming effect, we used a blocked design, where the 240 control condition trials always appeared before the 240 test condition trials. Within each condition, trials for each observability level were intermixed in randomized order.

#### Results

**Figure 5:**
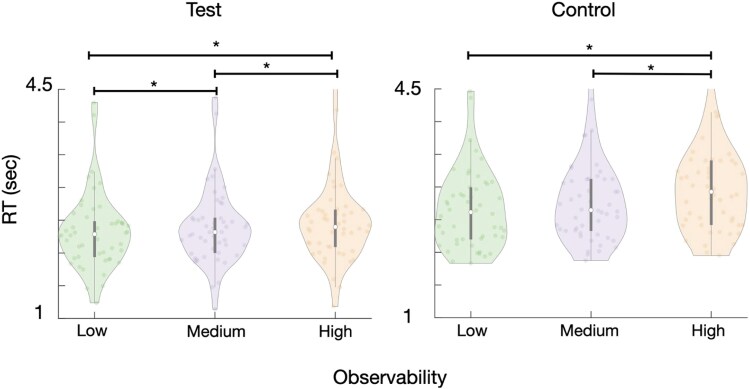
Experiment 2 results. The same conventions as in Fig. 3 are used here.

Similar to Experiment 1, the linear mixed effects model showed a significant effect of observability on RTs ($\beta =0.03, SE=0.003,t(20966)=8.74,P<.001, BF>100$), such that higher observability levels were associated with longer RTs. Here, unlike the previous experiment, there was also an effect of condition, with control trials evoking longer RTs overall ($2.80\pm 0.17)$ compared with test condition ones $(2.39\pm 0.07,\beta =0.15, SE=0.012,t(20966)=12.271,P<.001, BF>100$). To test whether this effect reflected experience-based adaptation, we compared the mean RTs of the first and last ten trials of each block, and found, based on a one-sided paired t-test, that early trials indeed exhibited longer RTs for both test $\left(0.12\pm 0.32,t(49)=4.42,P<.001\right)$ and control $\left(0.44\pm 0.7,t(49)=1.69,P=.049\right)$ conditions. Also, here a significant interaction between observability and condition was found, yet—unexpectedly—with a larger effect of observability on control condition trials compared to test ones ($\beta =0.03, SE=0.004,t(20966)=6.46,P<.001, BF=12.78$). *Post hoc* comparisons using the Tukey HSD test showed that in the test condition, RTs were longer for the high observability stimuli $\left(2.46\pm 0.01\right)$ compared with the medium $\left(2.39\pm 0.01\right)$, which in turn were longer than the low observability ones $\left(2.33\pm 0.01\right)$. A similar order relation was found in the control condition trials, which, as previously mentioned, showed overall longer RTs: $\left(2.99\pm 0.02\right)$, $\left(2.77\pm 0.02\right)$, $\left(2.65\pm 0.02\right)$, for the high, medium, and low “observability” stimuli, respectively. The results are shown in Fig. 5.

#### Discussion

Despite the blocked design, Experiment 2 replicated the main result of Experiment 1, namely, increased RTs for higher observability levels in both test and control conditions. The longer RTs in the control trials appear to reflect perceptual learning, with participants becoming more accustomed to the CFS stimulation as the experiment progressed, leading to overall shorter RTs in the test block. Indeed, a previous study showed that training increases the likelihood of breaking CFS suppression (Mastropasqua et al. 2015). A similar training effect was also found here when inspecting RTs within each block, such that the last trials in the block yielded faster RTs compared with the first ones.

Thus, while this experiment also failed to find evidence for the effect of epistemic ambiguity on RT, it provided additional evidence for the potential role of symmetry in driving b-CFS latency, as both experimental conditions showed an increase in symmetry between the stimuli. This difference in symmetry might have overshadowed a potential difference in observability, which could still be found. Thus, to make sure the potential effect of observability is properly tested, we ran a final experiment in which the stimuli were explicitly designed to disassociate symmetry and observability, such that observability is manipulated within the same level of symmetry. We reasoned that if observability has any effect on suppression time, an effect should be found in Experiment 3. If, instead, the results were fully driven by symmetry, no effect should be found in that experiment.

## Experiment 3: decoupling observability and symmetry

### Methods

#### Participants

Participants consisted of 50 healthy volunteers (39 female), between the ages 18–35 $\left(23.9\pm 3.4\right)$. Performance accuracy for all participants was above the exclusion threshold of 75% ($M=98.9\%, SD=2.1\%)$.

#### Stimuli and apparatus

**Figure 6:**
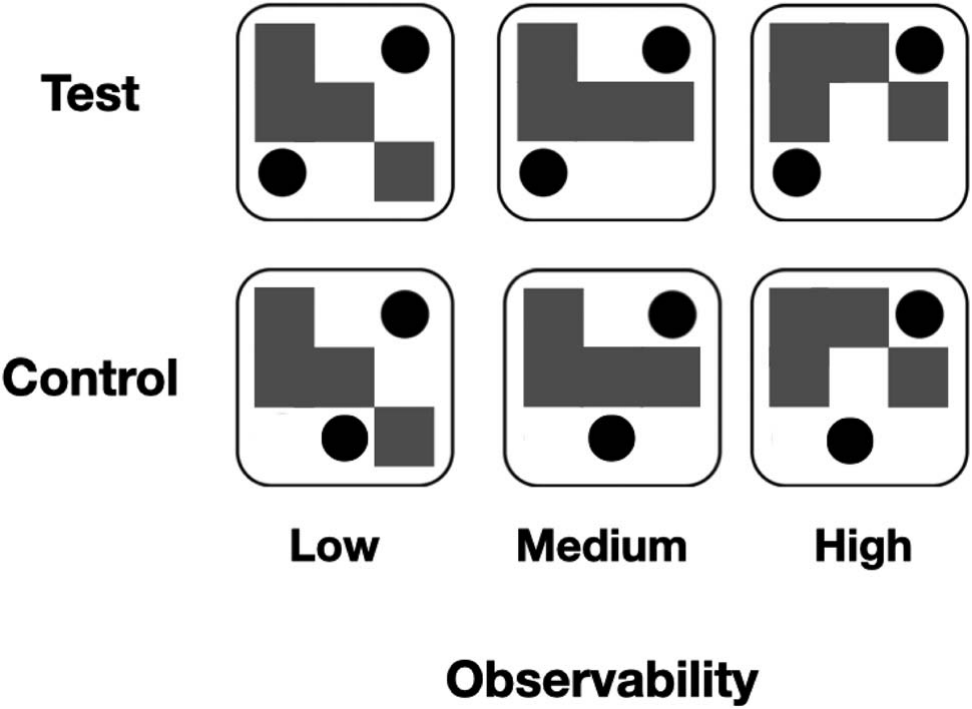
Experiment 3 stimuli: Test stimuli (top) were designed to have the same observability levels as in the previous experiments while lacking symmetry. Control stimuli (bottom) were designed to visually resemble the corresponding test ones while lacking semantic meaning.

The apparatus and the CFS stimulation were identical to the one used in Experiment 1. The test stimuli still consisted of partially occluded dice faces. Yet here, to tease apart symmetry and observability, the images were occluded by an asymmetric pattern of square “windows” (Fig. 6) instead of an occluding bar. Thus, across all observability levels, the stimuli were non-symmetrical for all axes. In the control stimuli, the position of the dots was shifted such that the stimuli could not be interpreted as standard dice configurations.

#### Procedure

The experimental procedure was similar to that of Experiment 1, other than the difference in dice stimuli. Because in Experiment 2 we found effects of observability levels also in the control condition, we reasoned that these did not stem from the mixed-trials within-participants design. Thus, to avoid an order effect, we returned to a fully randomized trial order across both conditions and all observability levels.

#### Results

**Figure 7:**
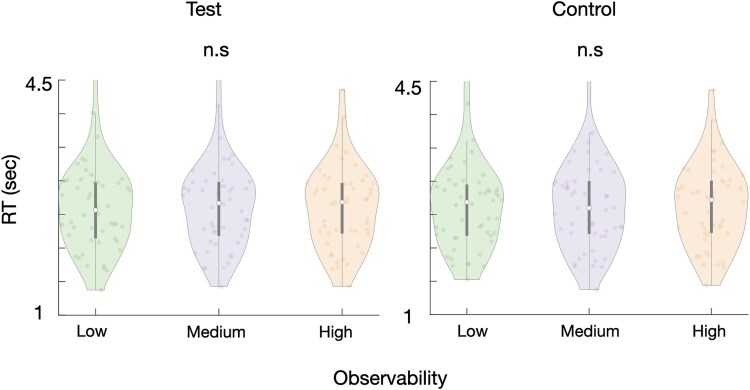
Experiment 3 results. The same conventions as in Fig. 3 are used here.

In this experiment, none of the results turned out significant: The linear mixed effects model described in Experiment 1 revealed no effect of observability on RTs ($\beta =0.001, SE=0.003,t(25196)=0.41,P=.681, BF<0.001).$ The effect of condition was also not significant, with control trial RTs $\left(2.62\pm 0.007\right)$ similar to those of test trials $(2.61\pm 0.01,\beta =0.006, SE=0.004,t(25196)=1.69,P=.273,$  $BF<0.001)$. The interaction between observability and condition was not significant either ($\beta =0.001, SE=0.004,t(25196)=0.45,P=.681, BF<0.001$). The results are shown in Fig. 7.

#### Discussion

Whereas in the previous two experiments observability and symmetry were (inversely) correlated, such that lower observability stimuli were also more symmetric, in this experiment, we addressed this potential confound by using strictly asymmetric stimuli with similar observability properties to those in the previous experiments. The lack of a significant difference in b-CFS RTs, in both test and control conditions, suggests that it was indeed the stimuli’s visual symmetry properties, rather than their observability, that drove the effects in Experiments 1 and 2.

### General discussion

In a series of three psychophysical experiments, we set out to test whether epistemic ambiguity, quantified by the control-theoretic notion of observability, facilitates access into visual awareness, as indexed by b-CFS RTs. Whereas experiments 1 and 2 showed an effect of observability on b-CFS RTs, this effect occurred in both test and control conditions, suggesting that it might be driven by mid-level perceptual features of the stimuli, like their symmetry levels, rather than their epistemic ambiguity. We therefore ran an additional experiment where observability and visual symmetry were dissociated, with the aim of manipulating only observability while keeping symmetry fixed. This third experiment did not find evidence for an effect of observability on interocularly suppressed stimuli.

Taken together, our results do not support the original hypothesis that epistemic ambiguity affects conscious access. Instead, *post hoc* analyses provide evidence that the mid-level perceptual features of the stimuli, possibly their symmetry levels, may affect emergence times, such that more symmetrical stimuli emerge faster into awareness. These findings are in line with previous studies suggesting that symmetry detection is highly efficient, versatile, and robust and that it occurs already at early processing stages (see Wagemans 1998 for a review). For example, an early study by Carmody et al. (1977) showed that symmetries can be detected efficiently even with very brief presentations (25 msec.). More recently, several studies using eye tracking measures found that eye fixations are strongly, and seemingly automatically, drawn to image regions that exhibit mirror symmetry (Kootstra et al. 2011, Meso et al. 2016). Interestingly, in a study testing symmetry detection in a patient with visual hemineglect, the patient exhibited robust symmetry sensitivity during an implicit detection test but failed when explicitly asked to report symmetry (Driver et al. 1992). Visual symmetry processing is known to recruit extended and automatic neural activation in the ventral stream (Bertamini et al. 2018). These findings suggest that symmetric stimuli are readily processed, enjoy preferential processing, and that they might be processed pre-attentively. Accordingly, this seems like a plausible interpretation of our results, though at this point it is still speculative, since our experiments were not designed to test it.

Several explanations have been proposed for this saliency of symmetry detection in the human visual system. First, symmetry is a factor in figure-ground and grouping, so it is directly linked to the perception of a good Gestalt (Wertheimer 1938). Symmetric stimuli may also be more readily processed by the brain since they are simpler—one part of a stimulus can be extrapolated from another. Hence, they require less cognitive effort to process (Attneave 1954). Second, from an evolutionary perspective, symmetry may be preferred as it is often associated with biological health and fitness. For example, symmetric facial features were found to elicit higher ratings of attractiveness (Grammer and Thornhill 1994). Symmetry detection has also been documented in other species, such as bees and spiders, showing clear preference for flowers that exhibit symmetry patterns (Wignall et al. 2006).

Can this natural tendency to favor symmetrical designs also facilitate access to awareness? While our results are consistent with this prospect (albeit only as a post-hoc interpretation), they do not necessarily imply that the evaluation of symmetry levels occurs *without visual awareness*, as the b-CFS paradigm has been criticized for the inability to determine if emergence times differ due to unconscious processes or post-perceptual, decision-related ones (Stein et al. 2011). Thus, we cannot draw that conclusion based on our findings. Future studies could explore this issue further, especially given a recent study that argued for the necessity of visual awareness for mirror symmetry-based grouping (Devyatko and Kimchi 2020). Notably, though, that study used a masked priming paradigm, whereas we used the b-CFS one. Although masking has been argued by some to allow less high-level processing than CFS (Breitmeyer 2015), others suggested that CFS actually operates on the earliest levels of the visual cortex (Yuval-Greenberg and Heeger 2013) or denied that it allows for integration to take place (Moors et al. 2016, though see Sklar et al. 2018). Thus, our positive findings for the effect of mid-level perceptual features (most likely, symmetry)—arguably requiring some level of integration in order to be deciphered—are of relevance to that debate as well, and suggest that at least some integration is possible under CFS, either during the suppression period, when the stimulus is unconsciously processed, or during the very last stages of suppression, just prior to emergence.

The lack of effect for observability is also relevant to the ongoing controversy about the level of processing under CFS. Our failure to find evidence for the influence of epistemic ambiguity on b-CFS emergence times may seem at odds with previous studies demonstrating b-CFS effects for related high-level stimuli features such as predictability (Pinto et al. 2015) or familiarity (Jiang et al. 2007). Our results may also seem at odds with a previous study that found evidence for an early (120 ms) occipital event-related potential signal correlated with perceptual disambiguation of an ambiguous Necker Cube figure (Kornmeier and Bach 2005b), suggesting that disambiguation may occur before perceptual awareness is established. However, unlike the perceptual ambiguity of the Necker Cube, the ambiguity in our experiment was meaningful, as the opposing alternatives imply different numerical values, possibly recruiting higher-level processes (Kouider and Dehaene 2009, Zerweck et al. 2021). In addition, even if disambiguation does occur early, this does not necessitate that it also affects emergence times. Overall, our findings are accordingly in better agreement with claims that the b-CFS paradigm is more affected by lower-level factors than by higher-level ones (Gayet et al. 2014, Moors et al. 2016). Furthermore, the lack of evidence for an effect of observability on b-CFS latency suggests that epistemic ambiguity resolution may occur only at the conscious processing stage, and not earlier, during unconscious processing or emergence to awareness. This result is supported by a recent study (Tal and Mudrik 2024), where invisible scenes did not disambiguate a subsequent visible object (while visible scenes did; though see (Biderman et al. 2020) for a study where invisible contextual cues disambiguated a visible ambiguous stimulus). Thus, more research is needed to better understand the relations between consciousness and ambiguity.

One could further speculate about the underlying computational mechanisms shaping the effect of symmetry levels on emergence into awareness. A possible clue toward elucidating these mechanisms is offered by an early study showing that polygons with multiple axes of symmetry are detected faster compared to ones with only a single axis (Palmer and Hemenway 1978). The researchers interpreted these findings as supporting a two-stage model of symmetry detection in which observers initially detect all symmetry axes in a crude, but rapid, parallel process and then perform a detailed sequential evaluation of each identified axis. More recently, behavioral, computational and electrophysiological studies have confirmed that the number of symmetry axes a stimulus exhibits is monotonically related to its perceived goodness (i.e. how easily is it judged to be processed and recognized) and simplicity (van der Helm and Leeuwenberg 1996, Hamada et al. 2016), as well as the neural response it evokes (Makin et al. 2016). Taken together, these findings support the notion that the number of symmetry axes exhibited by a stimulus is a key factor in determining its salience, which in turn may explain our results relating more symmetry axes with faster emergence from interocular suppression.

If so, what are the underlying computational mechanisms that can potentially explain our findings? One seemingly plausible hypothesis is that b-CFS latencies reflect the outcome of multiple evidence accumulation processes running in parallel (Smith and Vickers 1988, Usher and McClelland 2001), each corresponding to the detection of a different symmetry axis. Once sufficient evidence for the detection of a particular symmetry axis has been accumulated, the stimulus breaks suppression and can undergo higher-level processing (de Lange et al. 2011, Pereira et al. 2022). Such a model reflects the notion that the more symmetries are captured in an image, the less information from that image is needed to process it (Attneave 1955). Hence, in visually poor situations, with only little information about the stimulus available for processing, an object could be more easily computed from a more symmetric stimulus than from a less symmetric one. Future theoretical and empirical work could formulate and test the predictions of such a model of parallel symmetry detection under visual suppression.

Notably, however, there are several alternative interpretations to our findings that must be considered. First, with respect to the negative results we found regarding ambiguity, given the novelty of our manipulation of this factor and the lack of any independent effect of ambiguity, one might conclude that our paradigm simply failed to successfully manipulate semantic ambiguity. Under this account, epistemic ambiguity could still influence suppression times, yet here it did not do so since the stimuli did not effectively evoke ambiguity processing to begin with. Though we completely agree that this is a possible interpretation, we are not sure it is a plausible one, because participants were explicitly explained about the stimuli and their meaning, and their understanding was confirmed during the practice trials. Post-experiment debriefing also did not indicate in any way those participants failed to understand the ambiguity of the stimuli.

Second, doubts can also be raised with respect to the theoretical meaning of our positive results. While we interpret the effects as representing mid-level perceptual features of the stimuli, and focus mainly on symmetry, alternative accounts can be suggested. This concern is even more relevant since our study was not designed to test symmetry and accordingly did not include the relevant controls. For example, one might claim that the amount of occlusion or the centrality of the occluding bar is the driver of our effects. This could indeed be the case, although again, we are not sure that these explanations are more likely than the symmetry one. In experiments 1 and 2, occlusion levels are essentially identical in the medium and high observability stimuli, but there was still a consistently significant difference in the corresponding b-CFS latencies. Finally, symmetry detection has been established as a highly efficient, versatile, and robust perceptual mechanism that occurs already at early processing stages (see Wagemans 1998 for a review), making it a more likely interpretation of our results. However, even if the results are driven by the suggested factors and not by symmetry, the overall conclusion remains the same: mid-level perceptual factors affect emergence times. Future studies, planned ex-ante to test the effect of symmetry and other mid-level features, could arbitrate between these interpretations.

In conclusion, our findings do not support our original hypothesis that epistemic ambiguity facilitates access to awareness. They instead highlight the critical role of mid-level factors, most likely visual symmetry, in this process. Across three experiments, we observed that stimuli with higher levels of symmetry consistently enjoyed preferential access to awareness, suggesting that the brain’s inherent preference for symmetrical patterns may influence perceptual processing under suppression. These results align with previous studies demonstrating the perceptual advantages of symmetry, even at pre-attentive stages, and propose a potential mechanism involving parallel and rapid symmetry detection. While the lack of evidence for an ambiguity effect raises questions about the level of processing under b-CFS, it also leaves open the possibility that epistemic ambiguity resolution may occur only at later processing stages, or that its effects can be detected using other manipulations. Our work underscores the importance of disentangling mid-level visual properties from high-level epistemic factors in studying conscious access and encourages further exploration into how symmetry and ambiguity may interact in shaping perceptual awareness. Future research could address the proposed model of subliminal symmetry evaluation as a parallel evidence accumulation process, providing a deeper understanding of the mechanisms underlying the processing of stimuli exhibiting perceptual invariances and their emergence into awareness.

## Supplementary Material

OPEN_SCIENCE_BADGE_APPLICATION_FORM_niag006

Supplementary_material_mid_level_features_Dec_2_niag006
