# Supplementary material for: Mid-level perceptual features, and not ambiguity, accelerate access to awareness
Source: Neurosci Conscious. 2026 Feb 20;2026(1):niag006. doi: 10.1093/nc/niag006 (PMC12922541; doi:10.1093/nc/niag006)
Supplement: Supplementary_material_mid_level_features_Dec_2_niag006 [file supplementary_material_mid_level_features_dec_2_niag006.docx]

## Supplementary material


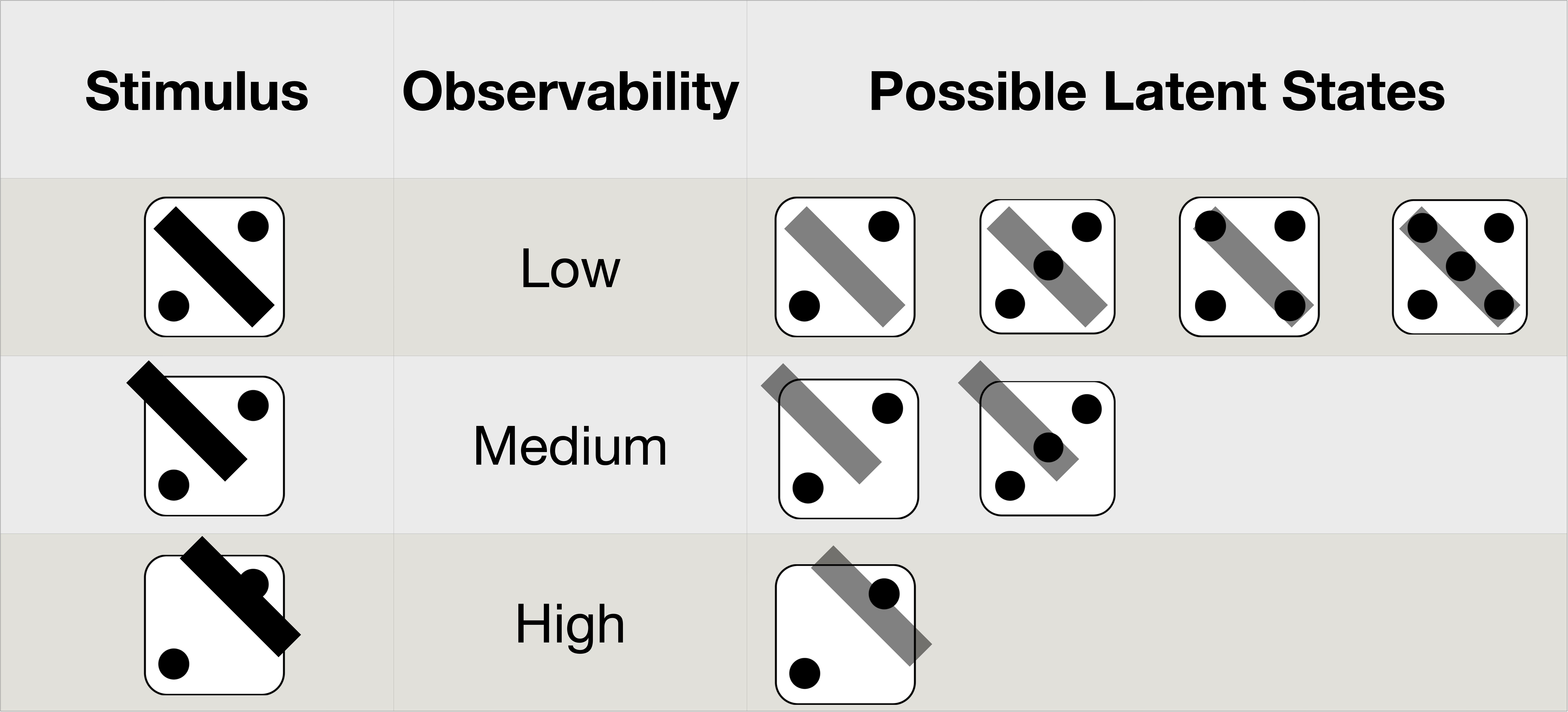


Figure S1: Possible interpretations of each stimulus in the test condition of Experiment 1 as partially occluded dice faces (latent states).


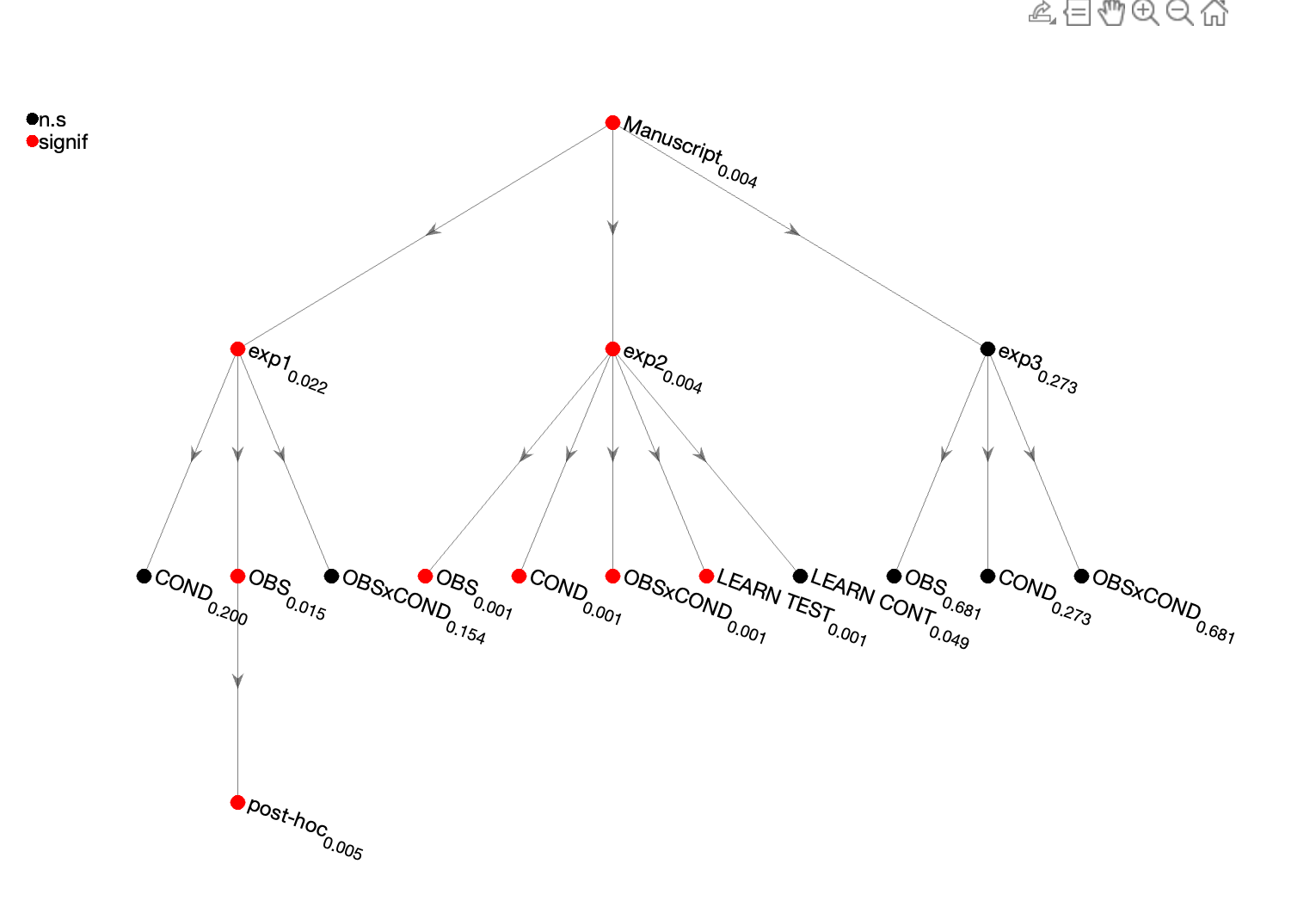


Figure S2 Tree structure for the analysis conducted in this study. Red nodes indicate results that remain significant after the TreeBH false discovery correction analysis.
